# Gender-Based Differences in COPD Patients with Type 2 Respiratory Failure—Impact on Clinical Practice

**DOI:** 10.3390/medicina61040587

**Published:** 2025-03-25

**Authors:** Tarkan Ozdemir, Murat Yıldız, Maşide Arı, Emrah Arı, Güler Eraslan Doğanay, Mustafa Özgür Cırık, Melek Doğancı, Çiğdem Özdilekcan, Derya Kızılgöz, Yusuf Tuğrul Şipit

**Affiliations:** 1 Department of Chest Diseases, Ankara Sanatoryum Training and Research Hospital, Ankara 06290, Turkey; drmuratyildiz85@gmail.com (M.Y.); masidetuten@icloud.com (M.A.); derya.ozaydin999@gmail.com (D.K.); yusuftugrulsipit@yahoo.com (Y.T.Ş.); 2 Department of Emergency Medicine, Mamak Public Hospital, Ankara 06620, Turkey; dremrahari25@gmail.com; 3 Department of Anesthesiology, Ankara Sanatoryum Training and Research Hospital, Ankara 06290, Turkey; gulerdoganay@hotmail.com.tr (G.E.D.); dr.ozgurr@hotmail.com (M.Ö.C.); melekdidik@hotmail.com (M.D.); 4 Department of Chest Diseases, Ankara Oncology Training and Research Hospital, Ankara 06200, Turkey; cigdemozdilekcan@yahoo.com.tr

**Keywords:** hypercapnia, NIV, BPAP, comorbidity, sex, female

## Abstract

*Background and Objectives:* To contribute to clinical practice by identifying gender-based differences in patients diagnosed with chronic obstructive pulmonary disease (COPD) who are monitored in the intensive care unit due to type 2 respiratory failure. *Materials and Methods:* The study was planned as a prospective, observational, and cross-sectional investigation. A total of 258 patients, 91 females and 167 males, were included in the study between 2023 and 2024. Demographic data and clinical parameters of COPD patients admitted to intensive care due to hypercapnic respiratory failure and treated with noninvasive ventilation (NIV) were compared between genders. *Results*: The number of male patients was higher than female patients, while the mean age of female patients was higher than that of males. The body mass index (BMI), morbid obesity, atrial fibrillation, renal disease, heart failure, hypertension, hypothyroidism, the Charlson Comorbidity Index (CCI), and the cardiothoracic ratio were found to be significantly higher in female patients. Emphysema and steroid use in treatment were more common in male patients. In laboratory analyses conducted at the time of admission, the average D-dimer and brain natriuretic peptide (BNP) levels were higher in female patients. The mean arterial carbon dioxide pressure (PaCO_2_) level assessed prior to discharge was also higher in female patients. *Conclusions*: Heart failure and risk factors that may lead to heart failure are more prominent in female COPD patients with type 2 respiratory failure. Despite the lower number of female patients compared to males, the significantly higher comorbidity burden in females, as per CCI scores, suggests that medical processes may be more challenging to manage in females. We believe that these findings will contribute to clinical practice and provide clinicians with insights for patient management.

## 1. Introduction

Chronic obstructive pulmonary disease (COPD) ranks among the leading causes of morbidity and mortality globally [1]. COPD is the third leading cause of death in the United States (US) [2]. The prevalence and burden of COPD are expected to increase in the coming decades due to continued exposure to risk factors and an aging global population [3]. The Global Initiative for Chronic Obstructive Lung Disease (GOLD) reports an overall prevalence of COPD at 11.8% in men and 8.5% in women [4], with significantly higher prevalence in men than women [5]. However, from 2001 to 2019, COPD prevalence has been steadily rising among women [6]. Although COPD is still primarily considered a “male disease”, mortality rates among men have been decreasing in some countries, such as the US and the United Kingdom, while remaining relatively unchanged among women [7,8].

Hypercapnic respiratory failure (HRF), also known as type 2 respiratory failure (T2RF), is characterized by an increase in the arterial carbon dioxide (CO_2_) pressure (PaCO_2_). CO_2_ levels in arterial blood are directly proportional to CO_2_ production and inversely proportional to alveolar ventilation. Increased dead space and decreased minute ventilation are common causes of hypercapnia [9]. During exacerbations, ventilation–perfusion (VA/Q) mismatches occur, potentially leading to arterial hypoxemia with or without hypercapnia [10]. Hypercapnia should always be suspected in individuals at risk of hypoventilation (e.g., sedative use, history of sleep apnea) or with increased physiological dead space and limited pulmonary reserve (e.g., COPD exacerbation) presenting with symptoms, such as dyspnea or altered mental status [9]. Acute or acute-on-chronic hypercapnia may develop during exacerbations or in response to oxygen therapy in some COPD patients [9]. Immediate admission to the intensive care unit (ICU) may be necessary for some cases. Ventilatory support during a COPD exacerbation can be provided via noninvasive (nasal or facial mask) or invasive ventilation [11]. Noninvasive ventilation (NIV) is the preferred first-line treatment for acute respiratory failure due to COPD exacerbations, with an 80–85% success rate in hypercapnic patients [12].

Many funding agencies in Europe and North America have implemented policies to encourage or mandate the consideration of sex and gender in medical research at all levels [13]. Despite significant advances in intensive care medicine, limited attention has been given to gender differences in the management and outcomes of ICU patients [14]. Although half of the global population comprises women, they account for only 35–45% of ICU patients [15]. Clinical presentations, comorbidities, and prognoses may differ by gender, potentially affecting treatment decisions [16].

A review of the literature reveals numerous studies on gender–COPD and gender–ICU topics. In these studies, multiple parameters, including demographic data, the severity of illness, comorbidities, Charlson Comorbidity Index (CCI), clinical and laboratory parameters, treatment responses, the need for mechanical ventilation, the length of hospital stay, mortality, and readmission rates, were compared among patients diagnosed with COPD or those followed in the ICU, stratified by gender, to identify similarities and differences [15,16,17,18,19,20,21,22,23,24,25,26,27,28,29,30].

However, no study has specifically compared COPD patients with T2RF treated with NIV based on gender. This study aims to identify gender-based differences in patients with HRF treated with NIV and to contribute findings to clinical practice.

## 2. Materials and Methods

### 2.1. Study Design and Inclusion and Exclusion Criteria

**Figure 1:**
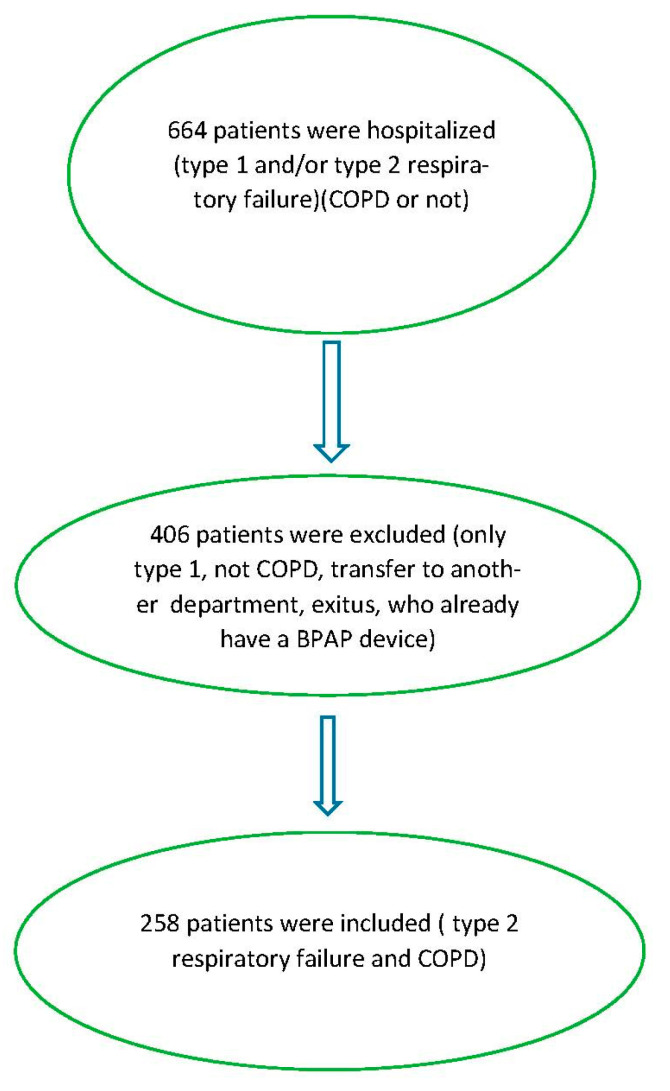
Flowchart of the study. COPD: chronic obstructive pulmonary disease.

Our study is prospective, observational, and cross-sectional. Since the study was observational, it was planned according to routine laboratory and radiologic examinations. Patients admitted to the second-level pulmonary ICU between 30 March 2023 and 30 March 2024 for T2RF and clinically stabilized with NIV before discharge were included. The hospital where the study was conducted is located in Ankara, the second largest city and capital of Türkiye. The estimated population of Ankara in 2025 is 5,909,658. The study was conducted in a university hospital with a capacity of 500 beds, which serves as a reference hospital for the pulmonology department. The secondary-level pulmonary intensive care department consists of two separate units with 11 and 12 beds, respectively. The patients included in the study were those followed in the 11-bed unit. There are a total of 14 NIV devices, 4 high flow oxygen devices, and 2 mechanical ventilator devices in the ward. The healthcare staff in the unit is trained and experienced in NIV application. There are 16 nurses working in shifts in the ward; 1 specialist physician and 2 assistant physicians work during the day, and 1 specialist physician and 1 assistant physician work on duty. In addition, to assist the nurses, 3 patient care personnel work during the daytime and 1 patient care personnel works during the on-call hours; 91 female and 167 male patients were enrolled in the study. Inclusion criteria were as follows: patients were over 40 years of age; patients had previously been diagnosed with COPD; patients had previously received a medication report for COPD medications; patients presented with hypercapnic respiratory failure. Exclusion criteria were as follows: included patients with type 1 respiratory failure (T1RF) alone; patients without a COPD diagnosis; those requiring intubation after NIV; patients transferred to third-level ICUs; COVID-19 PCR-positive patients transferred to COVID ICUs; patients who died during hospitalization for unrelated reasons; and patients with chronic hypercapnia previously using home BPAP. The inclusion and exclusion criteria of patients in the study were shown as a flowchart (Figure 1).

### 2.2. Data Collection and Study Protocol

Informed consent was obtained from patients prior to data collection. Patient files and the hospital information-management system served as data sources. In addition to demographic data, comorbidities, treatments received, long-term device use reports, length of stay, and routine laboratory parameters were recorded. To make a diagnosis of emphysema, new or previous thorax computed tomography scans of the patients were analyzed together with their reports through the radiological imaging system.

### 2.3. Descriptions

Standardized variables, such as the body mass index, Charlson comorbidity index, and cardiothoracic ratio, were used to compare the study groups.

Body Mass Index (BMI): BMI values were calculated as the ratio of height squared to body weight (kg/m^2^). The classification recommended by the World Health Organization was used: underweight below 18.5 kg/m^2^ was considered underweight, normal between 18.5–24.9 kg/m^2^, overweight between 25–29.9 kg/m^2^, obese above 30 kg/m^2^ and morbidly obese above 40 kg/m^2^ [31].

The Charlson comorbidity index (CCI) is one of the most commonly used methods to assess comorbid factors and predict mortality. The CCI takes into account many underlying conditions, such as age, kidney diseases, malignancies, cerebrovascular diseases, liver diseases, and lung diseases. Scoring is based on 19 medical conditions and is calculated based on variable morbidity rates in the patient population. The severity of morbidities is graded from 1 to 4 [32].

Cardiothoracic ratio: The cardiothoracic ratio (CTR), which expresses the relationship between the transverse size of the chest and the size of the heart measured on postero-anterior chest radiography, is a commonly used parameter in the evaluation of cardiomegaly and has a cut-off value of 0.5. A value greater than 0.5 should be interpreted as an enlarged heart [33].

Classification of disease groups was made according to the ICD 10 coding system used in Türkiye [34]: C34, malignant neoplasm of the bronchus and lung; D50, iron deficiency anemia; D51, vitamin B12 deficiency anemia; D52, folate deficiency anemia; D53, other nutritional anemias; E02, subclinical iodine-deficiency hypothyroidism; E03, other hypothyroidism; E66.2, extreme obesity with alveolar hypoventilation; E10, type 1 diabetes mellitus; E11, type 2 diabetes mellitus; F03, unspecified dementia; F20, schizophrenia; F41, other anxiety disorders; F41.2, anxiety depression; F32, depressive episode; G47.3, sleep apnea; G20, Parkinson disease; G30, dementia in Alzheimer disease; I10, essential (primary) hypertension; I25.1, atherosclerotic heart disease; I48, atrial fibrillation and flutter; I50, heart failure; I69, sequelae of cerebrovascular disease; I26, pulmonary embolism; J44.1, COPD with acute exacerbation; J96.0, acute respiratory failure; J47, bronchiectasis; J09-J18, pneumonia; J43, emphysema; M40.0, postural kyphosis; M40.1, other secondary kyphosis; M40.2, other and unspecified kyphosis; M41.3, thoracogenic scoliosis; N17, acute renal failure; N18, chronic kidney disease; N19, unspecified kidney failure; Q76.3, congenital scoliosis due to congenital bony malformation.

### 2.4. Statistical Analysis

The data were analyzed using the Statistical Package for Social Sciences (SPSS) Windows 27.0 software (Chicago, IL, USA). The normality of data distribution was evaluated using the Kolmogorov–Smirnov test and histograms. Numerical data with a normal distribution were presented as the mean ± standard deviation, while non-normally distributed numerical data were presented as the median and minimum–maximum values (IQR 25–75%). Categorical variables were expressed as numbers (n) and percentages (%). Categorical variables were compared using the Chi-square or Fisher’s Exact test, while continuous variables were compared using the Student’s *t*-test or Mann–Whitney U test. The Kruskal–Wallis analysis was used to compare more than two continuous variables. A *p*-value < 0.05 was considered statistically significant for all tests.

### 2.5. Ethical Considerations

Ethical approval for the study was obtained from the Ethics Committee of Ankara Sanatorium Training and Research Hospital (Date: 22 February 2023; No: 2012-KAEK-15/2644).

## 3. Results

Table 1 evaluates the gender distribution, ages, comorbidities, treatments, and hospital stay durations of the patients. The study was conducted with a total of 258 patients. Of these, 91 (35.3%) were female and 167 (64.7%) were male (*p* < 0.01). The mean age of the patients was 69 ± 10 years. The mean age of female patients was 72 ± 12 years, significantly higher than that of male patients (68 ± 8 years, *p* = 0.003) (Table 1). When comorbidities were compared by gender, body mass index (BMI) (*p* < 0.001), morbid obesity (*p* < 0.001), atrial fibrillation (*p* = 0.023), renal disease (*p* = 0.027), neurological diseases (*p* = 0.005), heart failure (*p* < 0.001), hypertension (*p* < 0.001), hypothyroidism (*p* = 0.024), CCI (*p* = 0.02), and CTR (*p* < 0.001) were significantly higher in female patients than in males (Table 2). Emphysema was significantly higher in male patients than in females (*p* < 0.001) (Table 1).

No significant differences were found in the gender comparison of patients prescribed long-term bilevel positive airway pressure (BPAP) at home (*p* = 0.730) or long-term oxygen therapy (LTOT) at home (*p* = 0.765) (Table 1).

When comparing treatments (antibiotics, steroids, antidepressants, anxiolytics, nutritional support), the number of male patients receiving steroid therapy was significantly higher than female patients (*p* = 0.004) (Table 1).

There were no significant differences in hospital stay durations between genders (*p* = 0.926) (Table 1).

Table 2 evaluates the laboratory parameters of the patients. In the analysis of laboratory parameters at admission, the average D-dimer value (*p* = 0.036) and brain natriuretic peptide (BNP) value (*p* = 0.04) were significantly higher in female patients than in males (Table 2). No significant differences were found in the comparison of pH compensation-decompensation in blood gas analysis at admission by gender (Table 2).

In the hemogram analyses conducted at admission and prior to discharge, the mean hemoglobin levels were significantly higher in male patients than in females (*p* < 0.001, *p* < 0.001) (Table 2).

In the blood gas analyses performed before discharge, the mean partial carbon dioxide pressure (PaCO_2_) (*p* = 0.036), actual bicarbonate (aHCO_3_) (*p* = 0.031), actual base excess (aBe) (*p* = 0.021), and standard base excess (sBE) (*p* = 0.035) values were significantly higher in female patients than in males. Additionally, in the biochemistry analysis, the mean sodium levels (*p* = 0.003) were significantly higher in female patients (Table 2).

Although not shown in the table, the lymphocyte/WBC ratio at admission, lymphocyte/WBC ratio at discharge, monocyte/WBC ratio at admission, monocyte/WBC ratio at discharge, neutrophil/WBC ratio at admission, neutrophil/WBC ratio at discharge, eosinophil/WBC ratio at admission, eosinophil/WBC ratio at discharge, basophil/WBC ratio at admission, and basophil/WBC ratio at discharge showed no significant differences between males and females (*p* = 0.25, *p* = 0.19, *p* = 0.34, *p* = 0.96, *p* = 0.52, *p* = 0.09, *p* = 0.75, *p* = 0.37, *p* = 0.89, *p* = 0.24, respectively). Additionally, no significant differences were found in blood group parameters (A, B, O, AB) when compared by gender (*p* = 0.92).

## 4. Discussion

Our study is the first to compare clinical parameters by gender in COPD patients treated with NIV and other medical therapies for T2RF in the ICU and subsequently discharged. The strength of the study is that it was conducted prospectively in an intensive care unit where a team experienced in NIV application worked and specialized in this field. The number of male patients was higher than that of females. The mean age of female patients was significantly higher than that of males. BMI, morbid obesity, atrial fibrillation, renal disease, heart failure, hypertension, hypothyroidism, CCI, BNP, BUN, D-dimer, CTR assessed on the first day of hospitalization, and PaCO_2_ measured before discharge were significantly higher in females. The presence of emphysema and the use of steroids in treatment were significantly more common in males. No significant gender differences were observed in patients prescribed long-term BPAP or oxygen therapy for home use.

A study examining the epidemiology of COPD in Türkiye using health insurance data found that 56.2% of physician-diagnosed COPD patients were male and 43.8% were female [35]. Another study on COPD exacerbations in Türkiye reported that 85% of the patients were male, while 15% were female [36]. Studies examining ICU patients have shown that the number of male patients is consistently higher [16,17,18,19,22,25,37]. In our study, consistent with the literature, the number of male patients was significantly higher than that of females.

A meta-analysis evaluating hospital admissions due to COPD exacerbations showed that the gender distribution varied widely, with mean ages ranging from 63.0 ± 14.5 to 76.3 ± 10.6 years [30]. Studies by Zetterstein et al. on ICU patients and Grabicki et al. on COPD patients found no differences in the mean age between males and females [16,25]. However, Todorov et al. reported a significantly higher mean age in female ICU patients than in males [75 (64;82) years in women vs. 68 (58;77) years in men, *p* < 0.001] [28]. In our study, the mean age of females was significantly higher than that of males. The life expectancy at birth in Türkiye is 80 years for women and 74.7 years for men, which may have influenced the results [38].

Comorbidities are common in all stages of COPD, from mild to severe [1]. A study evaluating COPD patients by gender found that the comorbidity burden, based on the CCI, was higher in women with severe and very severe COPD [26]. A study on ICU patients found fewer comorbidities in females than in males (5.4 vs. 6.4, *p* = 0.002) [26]. Among four studies examining CCI by gender, three found no significant differences [19,39,40], while one reported a higher comorbidity burden in males [25]. In our study, CCI was higher in females than in males. The higher average age in female patients compared to males is believed to impact the CCI, both directly due to age and indirectly due to the increased burden of comorbidities associated with aging.

Cardiovascular diseases are common and significant comorbidities in COPD [1]. The prevalence of systolic and diastolic heart failure in COPD patients ranges from 20% to 70% [41]. COPD is frequently associated with cardiac arrhythmias, which contribute to increased dyspnea [42]. Atrial fibrillation is common in COPD and can exacerbate dyspnea [43]. Hypertension is likely the most common comorbidity in COPD and can significantly impact disease progression [44]. Grabicki et al. found that cardiovascular diseases were more prevalent in females, while coronary artery disease was more common in males [16]. A study conducted in northern Sweden on COPD patients found a higher prevalence of cardiovascular disease in males [45]. Roche et al. found no gender differences in hypertension, ischemic heart disease, or left heart failure [21]. Todorov et al. reported no significant differences between genders in arrhythmias and heart failure [28]. In our study, heart failure, hypertension, atrial fibrillation, increased CTR (a radiological indicator of heart failure), and elevated BNP (a laboratory marker of heart failure) were significantly more common in females. No significant differences were observed between genders in coronary artery disease. It is thought that the higher prevalence of heart failure in women may be related to the frequency of hypertension and atrial fibrillation.

Patients with pulmonary etiologies and increased BMI are considered at risk for ICU admission [46]. Pulmonary causes are the most frequent reasons for hospital admission in patients with an elevated BMI [47]. A study by Kumar et al. on ICU patients found that among patients with a BMI of 30–39, 51.4% were male and 48.6% were female; for a BMI of 40–49, 41.5% were male and 58.5% were female; for a BMI ≥50, 41.9% were male and 58.1% were female [46]. Grabicki et al. found that females had lower BMIs than males [16]. In contrast, Roche et al. reported higher BMIs in females with COPD but no gender differences in the obesity prevalence [21]. In our study, females had significantly higher BMIs than males, and the number of morbidly obese female patients was greater than that of males. A higher age and comorbid disease burden in female patients may contribute to mobility restrictions, which, in turn, may be associated with a higher BMI.

In our study, renal failure and hypothyroidism were significantly more common in females. Matera et al. reported a higher prevalence of renal disease in males [27], and Grabicki et al. found thyroid diseases to be more common in females [16].

When we evaluated the relationship between an increased BMI, hypothyroidism, renal failure, and heart failure, all three conditions were found to be associated with heart failure and an increased heart failure risk. Heart failure often coexists with reduced renal function, and the relationship between the two conditions is bidirectional [47]. Being overweight (BMI ≥ 25 kg/m^2^) and having obesity (BMI ≥ 30 kg/m^2^) independently increase the risk of heart failure [48]. Hypothyroidism is also a risk factor for heart failure [49].

Other laboratory parameters significantly higher in females were D-dimer levels assessed on admission and PaCO_2_ levels measured before discharge. Elevated D-dimer levels are known to correlate with renal failure [50]. The higher PaCO_2_ levels before discharge in females may be due to the higher BMI and obesity prevalence, making hypercapnia more challenging to control in this group. In males, parameters significantly higher than in females included steroid use and the presence of emphysema. Males and females may exhibit phenotypically different responses to tobacco smoke exposure, with males being more prone to the emphysematous phenotype and females to the airway-dominant phenotype [51]. A review of the literature revealed no studies comparing systemic steroid use between men and women. In our study, the higher prevalence of steroid use in men could be attributed to the prioritization of diuretic therapy in women due to the higher incidence of heart failure as a comorbidity, relegating the anti-inflammatory steroid therapy to secondary importance.

The findings of this study are expected to guide future research. Furthermore, larger, multicenter studies focusing on gender-based comparisons across different age groups would contribute more significantly to clinical practice.

## 5. Limitations

The study was conducted at a single center. As this was an observational study, only routinely evaluated parameters were examined. Additional tests (e.g., echocardiography, spirometry) could not be performed. Due to the poor general condition of patients at the time of admission, a detailed medical history could not be obtained. Instead, anamnesis was collected from patient relatives and/or previous medical records were reviewed. The smoking history (amount, duration, age of initiation), biomass exposure, GOLD stage, alpha-1 antitrypsin levels, and frequency of exacerbations were not available. Due to the limited sample size, comparisons could not be made based on age groups. Chest radiographs used in the evaluation of CTR were performed with portable devices under intensive care conditions. The weight and height values used in the BMI calculation were either based on information obtained from patients/relatives or estimated. In cases where current data are not sufficient for the parameters used in the calculation of CCI, old records were also examined and included in the calculation.

### Strength of the Study

The strength of the study is that it was conducted prospectively in an intensive care unit where a team experienced in NIV application worked and specialized in this field.

## 6. Conclusions

In our study examining gender-based differences in COPD patients treated with NIV for T2RF, heart failure and risk factors for heart failure were found to be more prominent in female patients. Consistent with the literature, although the number of female patients was lower than that of males, the significantly higher comorbidity burden in females based on CCI scores suggests that medical processes may be more challenging to manage in women. We believe that these findings will contribute to clinical practice and provide clinicians with valuable insights into patient management.

## Figures and Tables

**Table 1:** Comparison of comorbidities, treatments, and hospital stay durations by gender.

	General	Female	Male	*p*
n (%)	258 (100%)	91 (35.3%)	167 (64.7%)	<0.01
Age	69 ± 10	72 ± 12	68 ± 8	0.03
Kyphoscoliosis	8 (3.1%)	5 (5.5%)	3 (1.8%)	0.102
Morbid obesity	26 (10.1%)	17 (18.7%)	9 (5.4%)	<0.001
OSAS	13 (5%)	6 (6.6%)	7 (4.2%)	0.400
Hypertension	147 (57%)	65 (71.4%)	82 (49.1%)	<0.001
Diabetes Mellitus	81 (31.4%)	32 (35.2%)	49 (29.3%)	0.336
Coronary artery disease	52 (20.2%)	20 (22.0%)	32 (19.2%)	0.591
Atrial fibrillation	39 (15.1%)	20 (22.0%)	19 (11.4%)	0.023
Kidney disease	72 (27.9%)	33 (36.2%)	39 (23.3%)	0.027
Neurological disease	7 (2.7%)	6 (6.6%)	1 (0.6%)	0.005
Depression/anxiety	44 (17.1%)	17 (18.7%)	27 (16.2%)	0.609
Bronchiectasis	19 (7.4%)	3 (3.3%)	16 (9.6%)	0.065
Pneumonia	17 (6.6%)	8 (8.8%)	9 (5.4%)	0.294
History of previous tuberculosis	12 (4.7%)	2 (2.2%)	10 (6.0%)	0.168
Pulmonary embolism	16 (6.2%)	8 (8.8%)	8 (4.8%)	0.204
Heart failure	124 (48.1%)	59 (64.8%)	65 (38.9%)	<0.001
Lung cancer	22 (8.5%)	4 (4.4%)	18 (10.8%)	0.080
Hypothyroidism	18 (7.0%)	11 (12.8%)	7 (4.7%)	0.024
Emphysema	69 (26.7%)	5 (5.5%)	64 (38.3%)	<0.001
Anemia	65 (25.2%)	27 (29.7%)	38 (22.9%)	0.233
Body mass index (Mean ± SD)	28 ± 8	31 ± 9	27 ± 7	<0.001
Charlson comorbidity index (Mean ± SD)	5 ± 2	5 ± 2	4 ± 1	0.002
Cardiothoracic ratio (Mean ± SD)	0.56 ± 0.09	0.61 ± 0.07	0.53 ± 0.09	<0.001
Length of stay (Mean ± SD)	9 ± 5	10 ± 7	9 ± 7	0.926
Systemic steroid treatment	184 (71.3%)	55 (60.4%)	129 (77.2%)	0.004
Antibiotic treatment	222 (86%)	77 (84.6%)	145(68.8%)	0.625
Anxiolytic/antidepressant treatment	38 (14.7%)	14 (15.4%)	24 (14.4%)	0.827
Nutritional support	19 (7.4%)	6 (6.6%)	13 (7.8%)	0.727
Number of patients prescribed BPAPs at home	138 (53.5%)	50 (54.9%)	88 (52.7%)	0.730
Number of patients prescribed OCs at homes	91 (35.3%)	31 (34.1%)	60 (35.9%)	0.765

OSAS: obstructive sleep apnea syndrome, BPAP: bilevel positive airway pressure, OC: oxygen concentrator.

**Table 2:** Analysis of laboratory parameters by gender.

	General 258 (100%)	Female 91 (35.3%)	Male 167 (64.7%)	*p*
Compensation in Hospitalization				0.761
Compensated (PH = 7.35–7.45) n (%)	187 (72.5%)	67 (73.6%)	120 (71.9%)	
Decompensated (PH < 7.35) n (%)	71 (27.5%)	24 (26.4%)	47 (28.1%)	
Admission PH	7.29 ± 0.08	7.28 ± 0.07	7.29 ± 0.08	0.686
Discharged PH	7.46 ± 0.05	7.47 ± 0.04	7.46 ± 0.05	0.402
Admission PaCO_2_	79.1 ± 15.7	77.5 ± 15.2	79.8 ± 16.0	0.239
Discharged PaCO_2_	50.4 ± 7.7	51.6 ± 6.1	49.8 ± 7.4	0.036
Admission aHCO_3_	37.8 ± 7.7	37.0 ± 9.0	38.3 ± 6.9	0.160
Discharged aHCO_3_	36.1 ± 5.3	37.0 ± 5.3	35.7 ± 5.3	0.031
Admission aHCO_3_	31.9 ± 7.3	31.9 ± 8.0	31.9 ± 6.8	0.680
Discharged aHCO_3_	34.5 ± 4.9	35.3 ± 4.4	34.1 ± 5.1	0.038
Admission aBe	8.5 ± 7.6	8.2 ± 8.4	8.6 ± 7.1	0.679
Discharged aBe	11.0 ± 5.3	11.7 ± 4.3	10.6 ± 5.8	0.021
Admission sBe	11.3 ± 7.9	10.4 ± 8.7	11.8 ± 7.3	0.208
Discharged sBe	12.9 ± 6.3	13 ± 5	12 ± 6	0.035
Admission CRP	77 ± 88	62 ± 76	86 ± 94	0.086
Discharged CRP	24 ± 29	20 ± 22	25 ± 32	0.792
Admission BUN	59 ± 34	66 ± 35	55 ± 33	0.006
Discharged BUN	52 ± 25	52 ± 23	52 ± 25	0.848
Admission creatinine	1.15 ± 0.59	1.16 ± 0.52	1.15 ± 0.63	0.591
Discharged creatinine	0.94 ± 0.50	0.87 ± 0.27	0.98 ± 0.59	0.136
Admission sodium	138.6 ± 4.8	139.0 ± 4.2	138.4 ± 5.1	0.500
Discharged sodium	138.6 ± 3.4	139.4 ± 3.4	138.2 ± 3.4	0.003
Admission potassium	4.58 ± 0.78	4.56 ± 0.72	4.60 ± 0.81	0.237
Discharged potassium	4.19 ± 0.63	4.14 ± 0.58	4.21 ± 0.67	0.229
Admission chloride	96.5 ± 6.7	96.3 ± 6.3	96.7 ± 7.0	0.399
Discharged chloride	96.1 ± 4.3	95.9 ± 4.7	96.1 ± 4.1	0.683
Admission magnesium	2.03 ± 0.35	2.00 ± 0.35	2.04 ± 0.36	0.636
Discharge magnesium	1.95 ± 0.24	1.91 ± 0.26	1.96 ± 0.22	0.183
Admission calcium	8.76 ± 0.76	8.67 ± 0.85	8.81 ± 0.70	0.594
Discharge calcium	8.69 ± 0.60	8.73 ± 0.61	8.67 ± 0.60	0.501
Admission albumin	3.50 ± 0.52	3.48 ± 0.50	3.51 ± 0.54	0.518
Discharge albumin	3.16 ± 0.45	3.14 ± 0.39	3.17 ± 0.48	0.465
Admission leukocyte	10.92 ± 4.39	11.01 ± 4.27	10.87 ± 4.46	0.753
Dischage leukocyte	9.08 ± 3.41	8.68 ± 2.91	9.30 ± 3.64	0.294
Admission lymphocyte	1.27 ± 1.06	1.36 ± 0.98	1.22 ± 1.10	0.127
Discharge lymphocyte	1.36 ± 1.39	1.29 ± 0.73	1.40 ± 1.65	0.680
Admission monocyte	0.611 ± 0.254	0.601 ± 0.352	0.616 ± 0.376	0.695
Discharge monocyte	0.589 ± 0.254	0.573 ± 0.234	0.598 ± 0.265	0.618
Admission neutrophil	8.81 ± 3.94	8.78 ± 3.91	8.83 ± 3.97	0.827
Discharge neutrophil	7.05 ± 2.96	6.62 ± 2.76	7.29 ± 3.05	0.119
Admission eosinophil	0.082 ± 0.185	0.073 ± 0.118	0.087 ± 0.213	0.938
Discharge eosinophil	0.123 ± 0.154	0.144 ± 0.191	0.111 ± 0.129	0.485
Admission basophil	0.037 ± 0.035	0.036 ± 0.026	0.038 ± 0.039	0.599
Discharge basophil	0.028 ± 0.022	0.029 ± 0.024	0.027 ± 0.021	0.493
Admission hemoglobin	13.2 ± 2.6	12.1 ± 2.3	13.8 ± 2.6	<0.001
Discharge hemoglobin	12.2 ± 2.6	11.4 ± 2.2	12.7 ± 2.7	<0.001
Admission platelet	244 ± 92	253 ± 94	239 ± 91	0.162
Discharge platelet	233 ± 87	229 ± 90	234 ± 85	0.456
Admission procalcitonin	0.81 ± 4.90	0.35 ± 0.99	1.07 ± 6.06	0.510
Discharge procalcitonin	0.11 ± 0.27	0.10 ± 0.24	0.11 ± 0.28	0.822
Admission D-dimer	2497 ± 4667	3535 ± 6411	1933 ± 3255	0.036
Admission troponin	347 ± 1660	249 ± 697	401 ± 2005	0.702
Admission BNP	355 ± 519	469 ± 603	294 ± 458	0.004
Admission T4	0.98 ± 0.23	0.95 ± 0.21	0.99 ± 0.24	0.381
Admission TSH	2.05 ± 9.32	3.42 ± 15	1.27 ± 2	0.640

PaCO_2_: partial arterial carbon dioxide pressure, aHCO_3_: actual bicarbonate, aBe: actual base excess, sBe: standard base excess, BNP: brain natriuretic peptide, TSH: thyroid-stimulating hormone.

## Data Availability

The original contributions presented in this study are included in the article. Further inquiries can be directed to the corresponding author.

## References

[1] Venkatesan P. (2024). GOLD COPD Report: 2024 Update. Lancet Respir. Med..

[2] Hoyert D.L., Xu J.Q. (2011). Deaths: Preliminary data for 2011. Natl. Vital. Stat. Rep..

[3] Mathers C.D., Loncar D. (2006). Projections of global mortality and burden of disease from 2002 to 2030. PLoS Med..

[4] Lamprecht B., McBurnie M.A., Vollmer W.M., Gudmundsson G., Welte T., Nizankowska-Mogilnicka E., Studnicka M., Bateman E., Anto J.M., Burney P. (2011). COPD in never smokers: Results from the population-based burden of obstructive lung disease study. Chest.

[5] Menezes A.M., Perez-Padilla R., Jardim J.R., Muiño A., Lopez M.V., Valdivia G., Montes de Oca M., Talamo C., Hallal P.C., Victora C.G. (2005). Chronic obstructive pulmonary disease in five Latin Ameri-can cities (the PLATINO study): A prevalence study. Lancet.

[6] Marshall D.C., Al Omari O., Goodall R., Shalhoub J., Adcock I.M., Chung K.F., Salciccioli J.D. (2022). Trends in prevalence, mortality, and disability-adjusted life years relating to chronic obstructive pulmonary disease in Europe: An observational study of the global burden of disease database, 2001–2019. BMC Pulm. Med..

[7] Agusti A., Faner R. (2018). COPD beyond smoking: New paradigm, novel opportunities. Lancet Respir. Med..

[8] Lozano R., Naghavi M., Foreman K., Lim S., Shibuya K., Aboyans V., Abraham J., Adair T., Aggarwal R., Ahn S.Y. (2012). Global and regional mortality from 235 causes of death for 20 age groups in 1990 and 2010: A systematic analysis for the Global Burden of Disease Study 2010. Lancet.

[9] Feller-Kopman D.J., Schwartzstein Richard M., Stoller J.K., Geraldine F. (2022). The evaluation, diagnosis, and treatment of the adult patient with acute hypercapnic respiratory failure. UpToDate.

[10] Barberà J.A., Roca J., Ferrer A., Félez M.A., Díaz O., Roger N., Rodriguez-Roisin R. (1997). Mechanisms of worsening gas exchange during acute exacerbations of chronic obstructive pulmonary disease. Eur. Respir. J..

[11] National Institute for Health and Care Excellence (2018). Chronic Obstructive Pulmonary Disease in over 16s: Diagnosis and Management. https://www.nice.org.uk/guidance/NG115.

[12] Rochwerg B., Brochard L., Elliott M.W., Hess D., Hill N.S., Nava S., Navalesi P., Antonelli M., Brozek J., Members of The Steering Committee (2017). Official ERS/ATS clinical practice guidelines: Noninvasive ventilation for acute respiratory failure. Eur. Respir. J..

[13] Mauvais-Jarvis F., Bairey Merz N., Barnes P.J., Brinton R.D., Carrero J.J., DeMeo D.L., De Vries G.J., Epperson C.N., Govindan R., Klein S.L. (2020). Sex and gender: Modifiers of health, disease, and medicine. Lancet.

[14] Merdji H., Long M.T., Ostermann M., Herridge M., Myatra S.N., De Rosa S., Metaxa V., Kotfis K., Robba C., De Jong A. (2023). Sex and gender differences in intensive care medicine. Intensive Care Med..

[15] Modra L., Higgins A., Vithanage R., Abeygunawardana V., Bailey M., Bellomo R. (2021). Sex differences in illness severity and mortality among adult intensive care patients: A systematic review and meta-analysis. J. Crit. Care.

[16] Grabicki M., Kuźnar-Kamińska B., Rubinsztajn R., Brajer-Luftmann B., Kosacka M., Nowicka A., Piorunek T., Kostrzewska M., Chazan R., Batura-Gabryel H. (2019). COPD Course and Comorbidities: Are There Gender Differences?. Adv. Exp. Med. Biol..

[17] Valentin A., Jordan B., Lang T., Hiesmayr M., Metnitz P.G. (2003). Gender-related differences in intensive care: A multiple-center cohort study of therapeutic interventions and outcome in critically ill patients. Crit. Care Med..

[18] Reinikainen M., Niskanen M., Uusaro A., Ruokonen E. (2005). Impact of gender on treatment and outcome of ICU patients. Acta Anaesthesiol. Scand.

[19] Fowler R.A., Sabur N., Li P., Juurlink D.N., Pinto R., Hladunewich M.A., Adhikari N.K., Sibbald W.J., Martin C.M. (2007). Sex-and age-based differences in the delivery and outcomes of critical care. CMAJ.

[20] Mahmood K., Eldeirawi K., Wahidi M.M. (2012). Association of gender with outcomes in critically ill patients. Crit. Care.

[21] Roche N., Deslée G., Caillaud D., Brinchault G., Court-Fortune I., Nesme-Meyer P., Surpas P., Escamilla R., Perez T., Chanez P. (2014). Impact of gender on COPD expression in a real-life cohort. Respir. Res..

[22] Kristensen M.L., Vestergaard T.R., Bülow H.H. (2014). Gender differences in randomised, controlled trials in intensive care units. Acta Anaesthesiol. Scand..

[23] Liao K.M., Chen Y.C., Cheng K.C., Wang J.J., Ho C.H. (2018). Trends in intensive care unit admissions of COPD patients from 2003 to 2013 in Taiwan. Int. J. Chron. Obstruct. Pulmon. Dis..

[24] Hill A., Ramsey C., Dodek P., Kozek J., Fransoo R., Fowler R., Doupe M., Wong H., Scales D., Garland A. (2020). Examining mechanisms for gender differences in admission to intensive care units. Health Serv. Res..

[25] Zettersten E., Jäderling G., Bell M., Larsson E. (2020). Sex and gender aspects on intensive care. A cohort study. J. Crit. Care.

[26] Backman B.H., Virchow J.C., Lundbäck B. (2021). COPD in women—New results presented. Respir. Med..

[27] Matera M.G., Ora J., Calzetta L., Rogliani P., Cazzola M. (2021). Sex differences in COPD management. Expert Rev. Clin. Pharmacol..

[28] Todorov A., Kaufmann F., Arslani K., Haider A., Bengs S., Goliasch G., Zellweger N., Tontsch J., Sutter R., Buddeberg B. (2021). Gender differences in the provision of intensive care: A Bayesian approach. Intensive Care Med..

[29] Rogliani P., Cavalli F., Ritondo B.L., Cazzola M., Calzetta L. (2022). Sex differences in adult asthma and COPD therapy: A systematic review. Respir. Res..

[30] Ruan H., Zhang H., Wang J., Zhao H., Han W., Li J. (2023). Readmission rate for acute exacerbation of chronic obstructive pulmonary disease: A systematic review and meta-analysis. Respir. Med..

[31] (1995). Physical status: The use and interpretation of anthropometry. Report of a WHO Expert Committee. World Health Organ Tech. Rep. Ser..

[32] Charlson M.E., Pompei P., Ales K.L., MacKenzie C.R. (1987). A new method of classifying prognostic comorbidity in longitudinal studies: Development and validation. J. Chronic Dis..

[33] Danzer C.S. (1919). The Cardiothoracic Ratio. Am. J. Med. Sci..

[34] World Health Organization International Statistical Classification of Diseases and Related Health Problems 10th Revision. https://iris.who.int/handle/10665/246208.

[35] Özdemir T., Yilmaz Demirci N., Kiliç H., Koç O., Kaya A., Öztürk C. (2020). An epidemiologic study of physician-diagnosed chronic obstructive pulmonary disease in the Turkish population: COPDTURKEY-1. Turk. J. Med. Sci..

[36] Ozyilmaz E., Kokturk N., Teksut G., Tatlicioglu T. (2013). Unsuspected risk factors of frequent exacerbations requiring hospital admission in chronic obstructive pulmonary disease. Int. J. Clin. Pract..

[37] Samuelsson C., Sjoberg F., Karlstrom G., Nolin T., Walther S.M. (2015). Gender differences in outcome and use of resources do exist in Swedish intensive care, but to no advantage for women of premenopausal age. Crit. Care.

[38] Başara B.B., Aygün A., Özdemir T.A., Kulali B. (2023). Health Statistics Yearbook 2023.

[39] Hollinger A., Gayat E., Feliot E., Paugam-Burtz C., Fournier M.C., Duranteau J., Lefrant J.Y., Leone M., Jaber S., Mebazaa A. (2019). Gender and survival of critically ill patients: Results from the FROG-ICU study. Ann. Intensive Care.

[40] Shen H.N., Lu C.L., Yang H.H. (2011). Women receive more trials of noninvasive ventilation for acute respiratory failure than men: A nationwide population-based study. Crit. Care.

[41] Bhatt S.P., Dransfield M.T. (2013). Chronic obstructive pulmonary disease and cardiovascular disease. Transl. Res..

[42] Liu X., Chen Z., Li S., Xu S. (2021). Association of Chronic Obstructive Pulmonary Disease with Arrhythmia Risks: A Systematic Review and Meta-Analysis. Front. Cardiovasc. Med..

[43] Terzano C., Romani S., Conti V., Paone G., Oriolo F., Vitarelli A. (2014). Atrial fibrillation in the acute hypercapnic exacerbations of COPD. Eur. Rev. Med. Pharmacol. Sci..

[44] Divo M., Cote C., de Torres J.P., Paone G., Oriolo F., Vitarelli A. (2012). Comorbidities and risk of mortality in patients with chronic obstructive pulmonary disease. Am. J. Respir. Crit. Care Med..

[45] Sawalha S., Hedman L., Backman H. (2019). The impact of comorbidities on mortality among men and women with COPD: Report from the OLIN COPD study. Ther. Adv. Respir. Dis..

[46] Kumar S.I., Doo K., Sottilo-Brammeier J., Lane C., Liebler J.M. (2020). Super Obesity in the Medical Intensive Care Unit. J. Intensive Care Med..

[47] Szlagor M., Dybiec J., Młynarska E., Rysz J., Franczyk B. (2023). Chronic Kidney Disease as a Comorbidity in Heart Failure. Int. J. Mol. Sci..

[48] Mandviwala T., Khalid U., Deswal A. (2016). Obesity and Cardiovascular Disease: A Risk Factor or a Risk Marker?. Curr. Atheroscler. Rep..

[49] Ning N., Gao D., Triggiani V., Iacoviello M., Mitchell J.E., Ma R., Zhang Y., Kou H. (2015). Prognostic Role of Hypothyroidism in Heart Failure: A Meta-Analysis. Medicine.

[50] Robert-Ebadi H., Bertoletti L., Combescure C., Le Gal G., Bounameaux H., Righini M. (2014). Effects of impaired renal function on levels and performance of D-dimer in patients with suspected pulmonary embolism. Thromb. Haemost..

[51] Han M.K., Postma D., Mannino D.M., Giardino N.D., Buist S., Curtis J.L., Martinez F.J. (2007). Gender and Chronic Obstructive Pulmonary Disease: Why It Matters. Am. J. Respir. Crit. Care Med..

